# Game-Based E-Learning Is More Effective than a Conventional Instructional Method: A Randomized Controlled Trial with Third-Year Medical Students

**DOI:** 10.1371/journal.pone.0082328

**Published:** 2013-12-05

**Authors:** Martin Boeker, Peter Andel, Werner Vach, Alexander Frankenschmidt

**Affiliations:** 1 Department of Medical Biometry and Medical Informatics, University Medical Center Freiburg, Freiburg i. Br., Baden-Württemberg, Germany; 2 Center de Sandà, Sta, Maria, Grisons, Switzerland; 3 Department of Urology, University Medical Center Freiburg, Freiburg i. Br., Baden-Württemberg, Germany; Hungarian Academy of Sciences, Hungary

## Abstract

**Background:**

When compared with more traditional instructional methods, Game-based e-learning (GbEl) promises a higher motivation of learners by presenting contents in an interactive, rule-based and competitive way. Most recent systematic reviews and meta-analysis of studies on Game-based learning and GbEl in the medical professions have shown limited effects of these instructional methods.

**Objectives:**

To compare the effectiveness on the learning outcome of a Game-based e-learning (GbEl) instruction with a conventional script-based instruction in the teaching of phase contrast microscopy urinalysis under routine training conditions of undergraduate medical students.

**Methods:**

A randomized controlled trial was conducted with 145 medical students in their third year of training in the Department of Urology at the University Medical Center Freiburg, Germany. 82 subjects where allocated for training with an educational adventure-game (GbEl group) and 69 subjects for conventional training with a written script-based approach (script group). Learning outcome was measured with a 34 item single choice test. Students' attitudes were collected by a questionnaire regarding fun with the training, motivation to continue the training and self-assessment of acquired knowledge.

**Results:**

The students in the GbEl group achieved significantly better results in the cognitive knowledge test than the students in the script group: the mean score was 28.6 for the GbEl group and 26.0 for the script group of a total of 34.0 points with a Cohen's d effect size of 0.71 (ITT analysis). Attitudes towards the recent learning experience were significantly more positive with GbEl. Students reported to have more fun while learning with the game when compared to the script-based approach.

**Conclusions:**

Game-based e-learning is more effective than a script-based approach for the training of urinalysis in regard to cognitive learning outcome and has a high positive motivational impact on learning. Game-based e-learning can be used as an effective teaching method for self-instruction.

## Introduction

When compared with more traditional instructional methods, Game-based E-learning promises a higher motivation of learners by presenting content in an interactive, rule-based and competitive way. Activation of learners supports the learning process not only in the cognitive domain but also in the affective and psycho-motor domains [1,2]. Notwithstanding these high expectations, the evidence provided by studies in pre- and post-graduate medical education is limited and equivocal [3–6]. Two recent studies did show that electronic games could be an effective means of teaching medical content [7,8]. However sparse the existing evidence for better learning outcome, educational games could still be of exceptional value for the self-study of topics in which learners have motivational problems.

GbEl can combine the benefits of learning in a high-fidelity multi-medial and simulative learning environment with game-based learning approaches [9]. Electronic game-based learning has a long tradition in medical education with the release of first applications in the 1960s and has gained much attention in recent years due to rapid technical advances in computer and gaming industries [10]. A wide variety of studies and reviews in game-based learning in medicine is available for different application scenarios and user-groups, but only few were explicitly directed towards game-based electronic learning [7,11–14]. The most frequent game formats found in medical education are from the genres of popular card and quiz games [7,11,15–24].

A variety of electronic resources available for undergraduate and graduate medical education in urology go beyond the function of an interactive textbook. Most prominently, the American Urological Association (AUA) distributes a variety of e-learning programs and multimedia enhanced educational materials for different educational levels [25]. Provided by the AUA, UroChallenge is an electronic quiz game also available for different mobile devices. Different approaches to electronic (distance) learning have been successfully evaluated in undergraduate and graduate medical education in urology [26–28]. UroSurf, a teaching program of the University of Bern, Switzerland, includes a quiz function and is widely and successfully used in student training throughout Switzerland [29]. To date there is no literature evaluating the use of GbEl programs in urological curricula for medical students.

In this work, we addressed the problem that today only few randomized controlled trials on the effectiveness of GbEl on learning outcome are available, which have a sufficient methodological quality to be included in meta-analysis. Thus, the evidence on the efficacy and effectiveness of GbEl remains low [3–6,8], despite high expectations. With this study we sought to directly measure the impact of GbEl on the cognitive learning outcome under *everyday teaching conditions*, as well as the attitudes of students towards their learning experience. To our knowledge, this is the first RCT on the effectiveness of GbEl in urology and one of few RCTs on the effectiveness GbEl in medical education in general following a rigorous methodological approach.

The aim of this study was to show the superiority of Game-based E-learning to a conventional instructional method for medical students learning phase contrast microscopy of urine specimens. The hypothesis of this study was that the cognitive learning outcome of third-year medical students learning phase contrast microscopic urinalysis with an electronic educational adventure-game is better than those of students learning the same subject with a written script. Additional data on attitudes and satisfaction were collected, including enjoyment of the learning experience, desire for further learning of this style, and self-assessment of achieved knowledge.

## Methods

This randomized controlled trial was conducted in the winter-semester 2008/9 in the Urological Department at the Freiburg University Medical Center in Freiburg, Germany. It is reported in accordance with the CONSORT 2010 statement [30]. The study population consisted of medical students in their third year of education at the Freiburg University Medical Center, who participated in the urological curriculum.

The urological curriculum is composed of weekly lecture during the semester and a one-week practical training, in which the students rotate in small groups of 3-6 students through the urological department. By drawing lots, the Students' Dean Office randomly assigned the students to the week of the semester in which they were trained. All 145 third-year medical students participating in the training at the Department of Urology were eligible for inclusion in the study. At the beginning of each week of practical training, the students were told that they could optionally participate in an educational study and that their participation or non-participation would have no effect on their final results and grades.

One semester prior to the study, we validated the measurement instrument (see below) with a group of students who had not received the specific training of the intervention. This group is referred to as the *reference group* and consisted of 117 students just before the urological curriculum, who had otherwise received the same instruction as the two study groups.

### Student Allocation

Power analysis was done prior to student allocation. A balanced group size of 59 in each arm was calculated for an expected moderate effect size (Cohen's d = 0.6) with a two-sided alpha = 0.05 and a power = 0.9.

**Figure 1:**
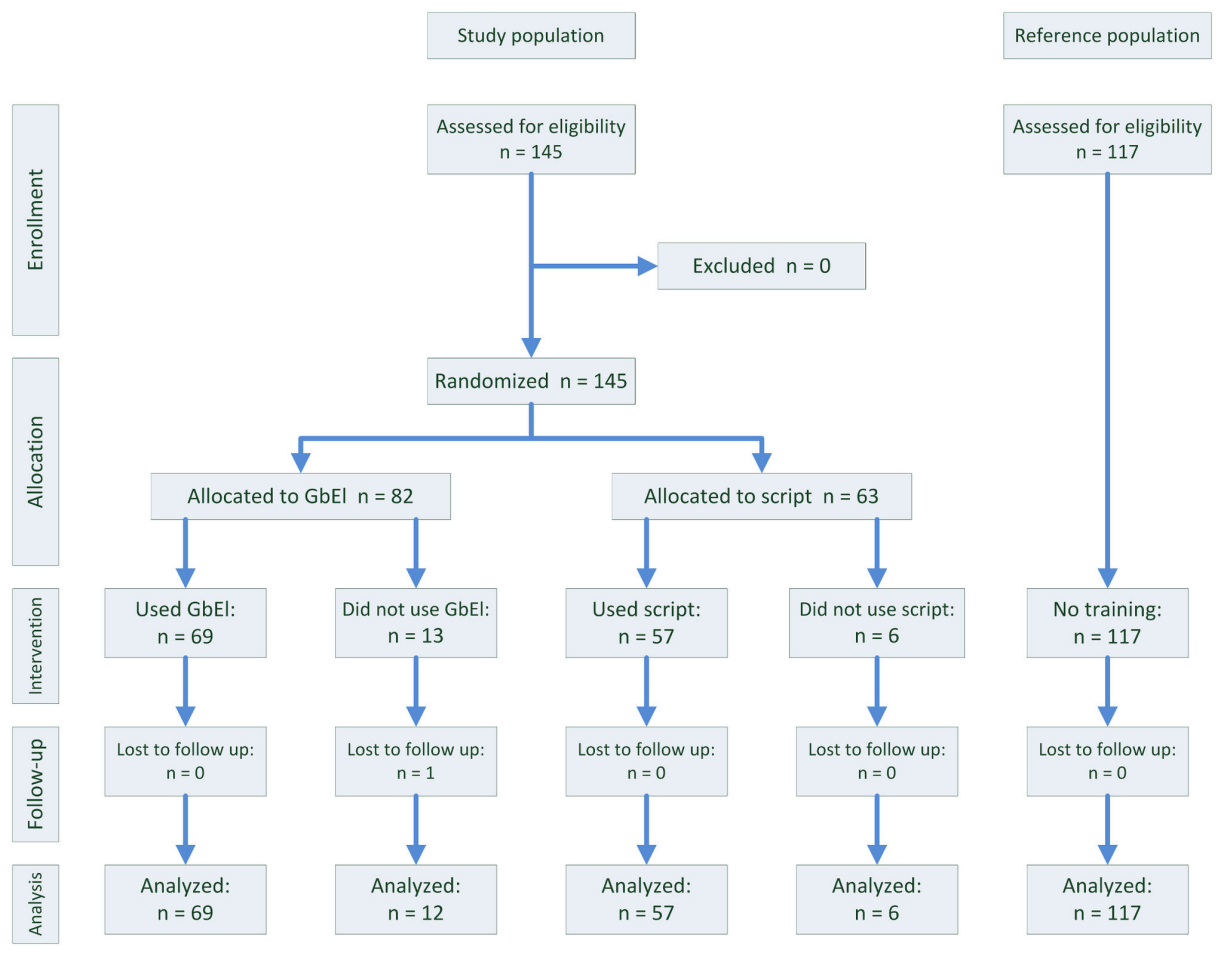
CONSORT Diagram. A total of 145 students were randomized of which 144 students were analyzed.

The allocation and randomization schema of the study was based on the random allocation of students to the *practical week* by the Students' Dean Office and the weekly change of the interventional method from script (control) to GbEl (intervention). The students were randomized for the entirety of the practical week, although the intervention affected only a small part of the practical week (one of six different workplaces). In this way, a *total of 145 students* where allocated alternately every other week either to the control or to the intervention arm of the study to equally distribute the effects of accumulating knowledge acquired during the progress of the lecture. 63 students were allocated to the control group (script) and 82 students to the intervention group (GbEl). Finally, 57 students of the control group and 69 students of the intervention group conducted the training they were randomized for: 6 students in the control group did not use the script and 13 students in the intervention group did not play the game. Of the latter, one student was lost to follow up, due to missing data in the questionnaire. The number of individuals in the control and intervention arms in the phases of the trial is displayed in the CONSORT diagram (Figure 1).

### Intervention

Students in the control group (script) learned the topic of "Phase contrast microscopic urinalysis on native urine" in a conventional script-based approach, while the students in the intervention group (GbEl) learned the topic with an electronic adventure-game.

For lectures and practical parts of the urology curriculum, both groups received exactly the same instructional contents and methods with the single exception of the instructional method of the intervention. For the script-based approach, the students were provided at the workplace with three exemplars of an 8-page script containing all the material to acquire knowledge on the learning objectives. The script includes an introductory part on the sampling of native urine and working on a phase contrast microscope. In the following section, the script describes bacteria, fungi, crystals, and corpuscular components of the urine like leucocytes, erythrocytes, and epithelial cells. Finally, the script explains diseases in which the microscopical findings are important for general practitioners as well as for urologists: cystinuria, bacterial urinary tract infection, asymptomatic bacteriuria, hematuria, sterile leukocyturia, and urolithic crystalluria.

For the intervention group, two PCs installed with the game described below were provided. No further written material was provided for the GbEl group. The learning objectives were identical for both groups.

The interventional electronic adventure game, Uro-Island, had been developed in the Department of Urology of the University Medical Center Freiburg in cooperation with the Institute of Medical Informatics and Medical Biometry, University of Freiburg in Freiburg, Germany [31]. It is built on the open source Wintermute game engine [32] under the GNU Lesser General Public License and can be played on computers with MS Windows operating system.

**Figure 2:**
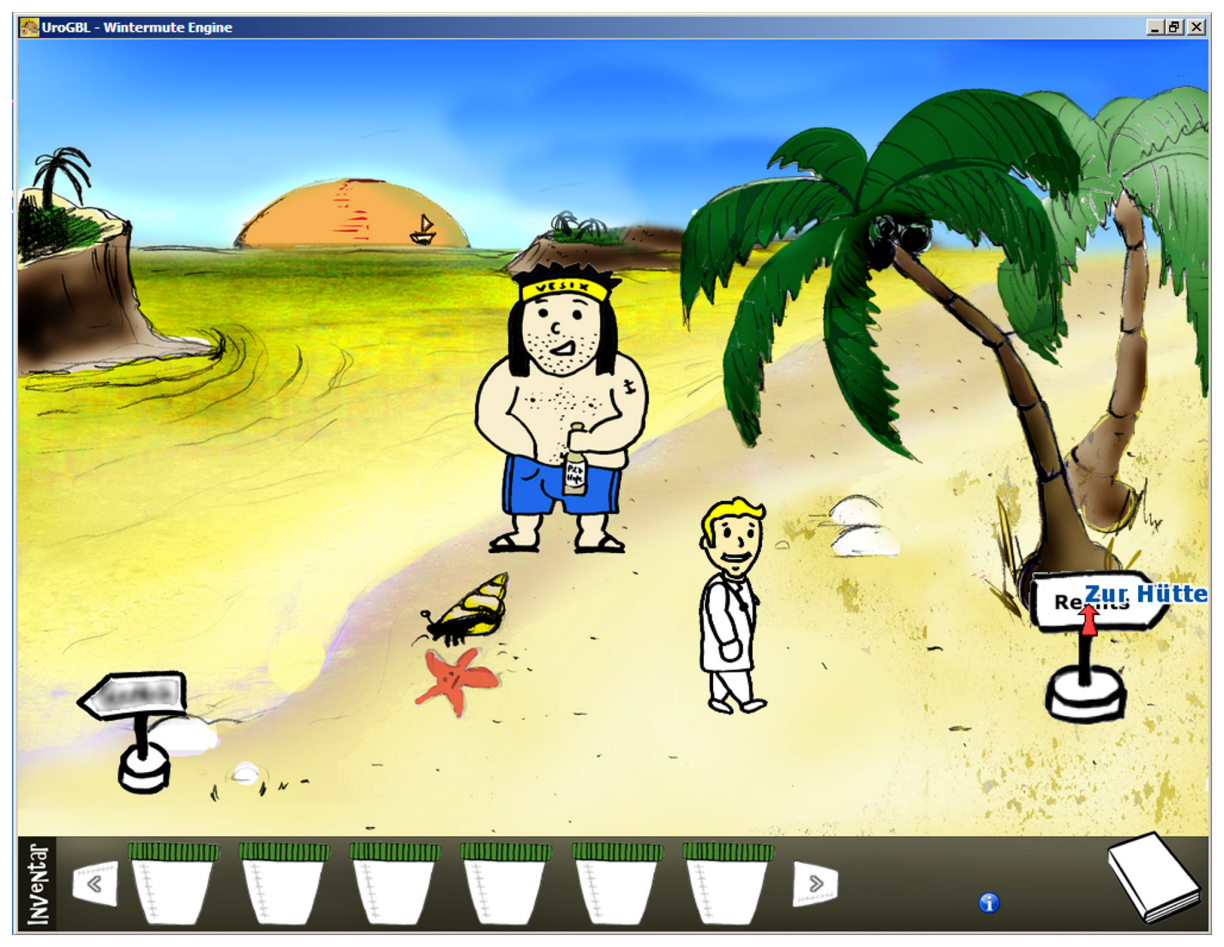
Scene from Uro-Island. The character controlled by the player ("the doctor") exploring the island. He engages in dialogues with the non-player character ("Vesix).

**Figure 3:**
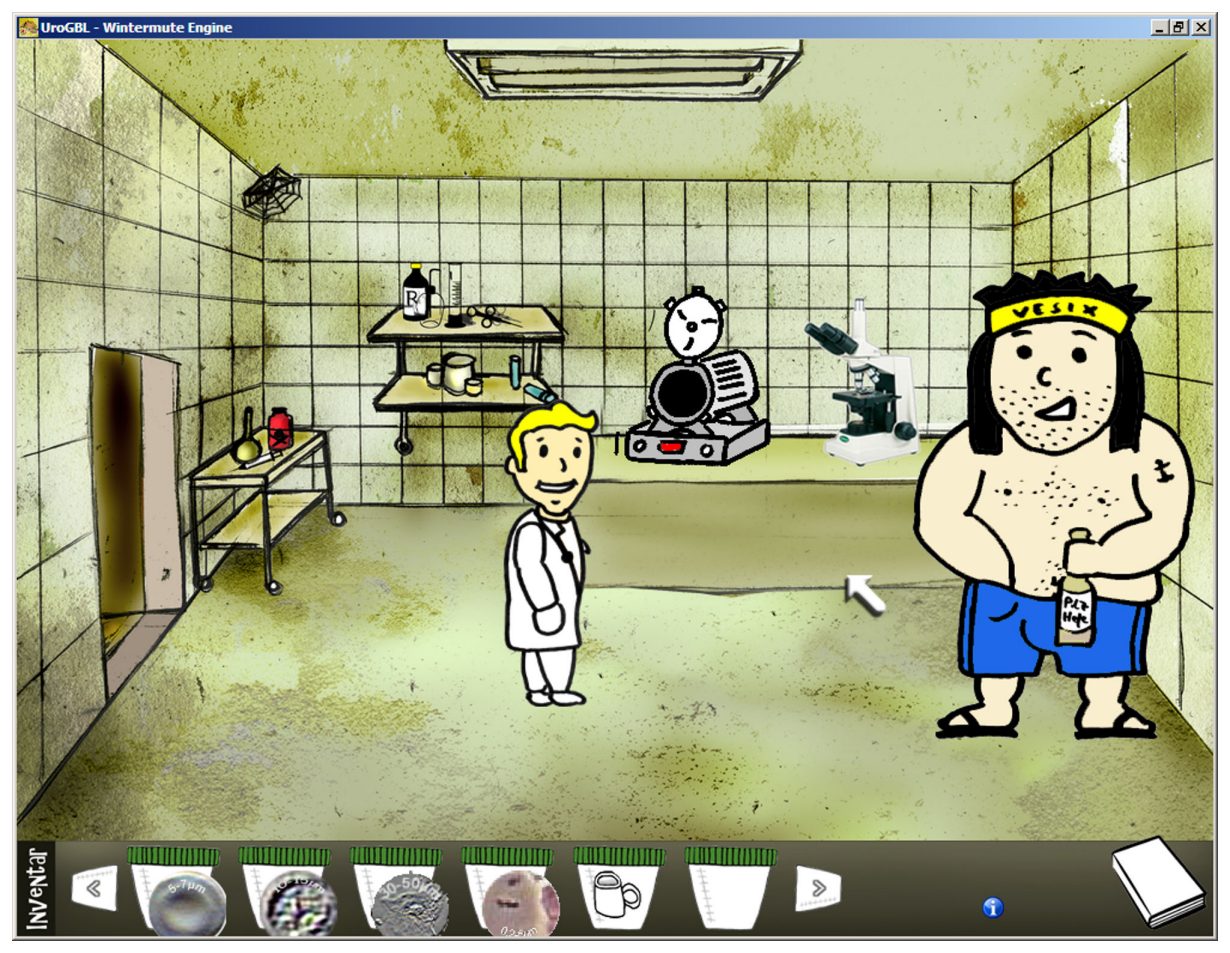
Scene from Uro-Island. The players' character together with Vesix in a laboratory. Different things like cells, cristalls and tools have been collected into the inventory. Later the character can use them together with the correct devices in the laboratory, e.g. the microscope or the autoclave, to proceed in the unfolding story of the game.

Characteristic of the adventure game genre, the player has to control and navigate the game character to explore a landscape with different embedded scenarios in order to eventually complete a series of quests [33,34]. During the unfolding story, the character can be controlled to engage in dialogues with non-player characters to gain information necessary to complete the quests (Figure 2). Another feature of adventure games is the gathering of items and attributes which are necessary to use in subsequent episodes of the story (Figure 3). In the case of Uro-Island, the female or male character, depending on the choice of the player, has to explore an Island on which his ship has stranded. The character has to master a series of quests, each related to exactly the same urine pathologies as described in the script. The ultimate goal is to gain the character's freedom back by advancing to a clear diagnosis and to finally leave Uro-Island.

Educational or serious games integrate and embed educational content into the game without destroying the nature of game [35,36]. In this way, the educational contents of Uro-Island, as defined by the educational objectives, are integrated in the story in separate modules (Table 1). Controlled by the player (learner), the character encounters the quests in increasing difficulty and adheres to a constituent internal structure, e.g. selection of the phase contrast microscope for urinalysis, corpuscular and cellular components of the urine, bacteria in the urine, and types of crystals in the urine and their dependence on the urine pH.

**Table 1:** Major cognitive learning objectives of the curriculum on "Phase contrast microscopic urinalysis on native urine".

**By the end of the curriculum, each student will be able …**	module
to describe the correct urine sampling method for enzymatic, microscopic and cultural urine diagnostics in urology	urine sampling
to describe the following methods of urine diagnostic: enzymatic urine test strip, preparation of a urine culture, and phase contrast microscopy of native urine	urine diagnostic methods
to describe the morphology of the pathological components in phase contrast microscopy of the native urine and name the pathological components	corpuscular elements, bacteria, crystals
to assign the common microscopic and enzymatic findings to urological diseases and interpret those findings bacte	corpuscular elements, ria, crystals

All learning objectives were subject both in the script based approach and the GbEl. The module in which the particular learning objective is mainly trained is allocated.

### Outcome measures and data collection

Primary outcome measure was the performance of students in achieving cognitive objectives of the curriculum on a program level. Cognitive educational objectives were defined prior to the curriculum development [37] (Table 1). Students' performance was measured with a written single-choice test designed to assess the specified educational objectives with 34 questions. Each correct answer was counted as one point. The true-false questions were iteratively developed [38] by two undergraduate students and three experienced urologists. The test was piloted on the reference group (n = 117), which consisted of students prior to the specific training who were otherwise on the same educational level as the study group. Cronbach's α for the test was α = 0.73 in the study group (n = 144). Cronbach's α is a measure for the internal consistency of a test. Values above 0.7 are an indicator for an *acceptable* internal consistency of a test [39].

Secondary outcomes were the attitudes students articulated in respect to the learning experience: (1) Did you enjoy working with the learning material?, (2) Would you like to have more learning material like this in your training?, and (3) Do you feel confident in the domain of laboratory urinalysis now?. Attitudes were measured with a questionnaire on a 4-point Likert scale.

Data was collected anonymously on forms in the debriefing session of the practical training week in urology. The data were collected by rotating physicians of the department in charge of the students' training in the practical week. Data were transferred to spreadsheets for further analysis.

### Statistical analysis

Item analysis and Cohen's kappa were computed with the R-statistical package. Stata was used to perform descriptive and test statistics, as well as instrumental variable regression analysis [40]. An intention-to-treat-adjusted-for-treatment (ITT-AT) analysis of the outcome measure was performed to correct the standard intention-to-treat (ITT) analysis for non-compliance. An ITT-AT analysis is a regressions analysis in which the influence of the treatment on the outcome is calculated under consideration of the randomization status as an instrumental variable. In settings where many study participants are not treated as randomized, an ITT-AT analysis can help to evaluate the true treatment effect more precisely than standard ITT analysis [41,42].

### Ethical approval

Ethical approval was requested from the ethical authority of the University of Freiburg, Freiburg, Germany. The chair of the University of Freiburg ethics committee reviewed the project and concluded that a full formal ethics committee statement was not required, due to the educational nature of the study. It was designed according to the general requirements for educational studies at the University Medical Center Freiburg, Freiburg, Germany, and was performed with the informed consent of the participants.

Before the training, students were orally informed about the nature of the study in which a new instructional method was investigated *to the benefit of the students* prior to its routine deployment. They were further informed that their participation was entirely voluntary, and that the topic used in the intervention of the study (urinalysis) was not part of the written examination after the training in that semester, so that a participation or non-participation would have no consequences on their grades. Students were further informed that they could use the other type of training they had not used in their training week directly after the study.

The assessment in the end of the training week was anonymous. With the participation in the assessment, the students' agreed on the anonymously analysis of their data. Students who would not like to participate could chose to deny participation without further consequences. Anonymous participation is document of the oral agreement of the students.

This type of orally agreement employed in our study was discussed with the ethics committee which agreed on it. Due to practical reasons, a formal written consent prior to the study was not feasible.

## Results

**Figure 4:**
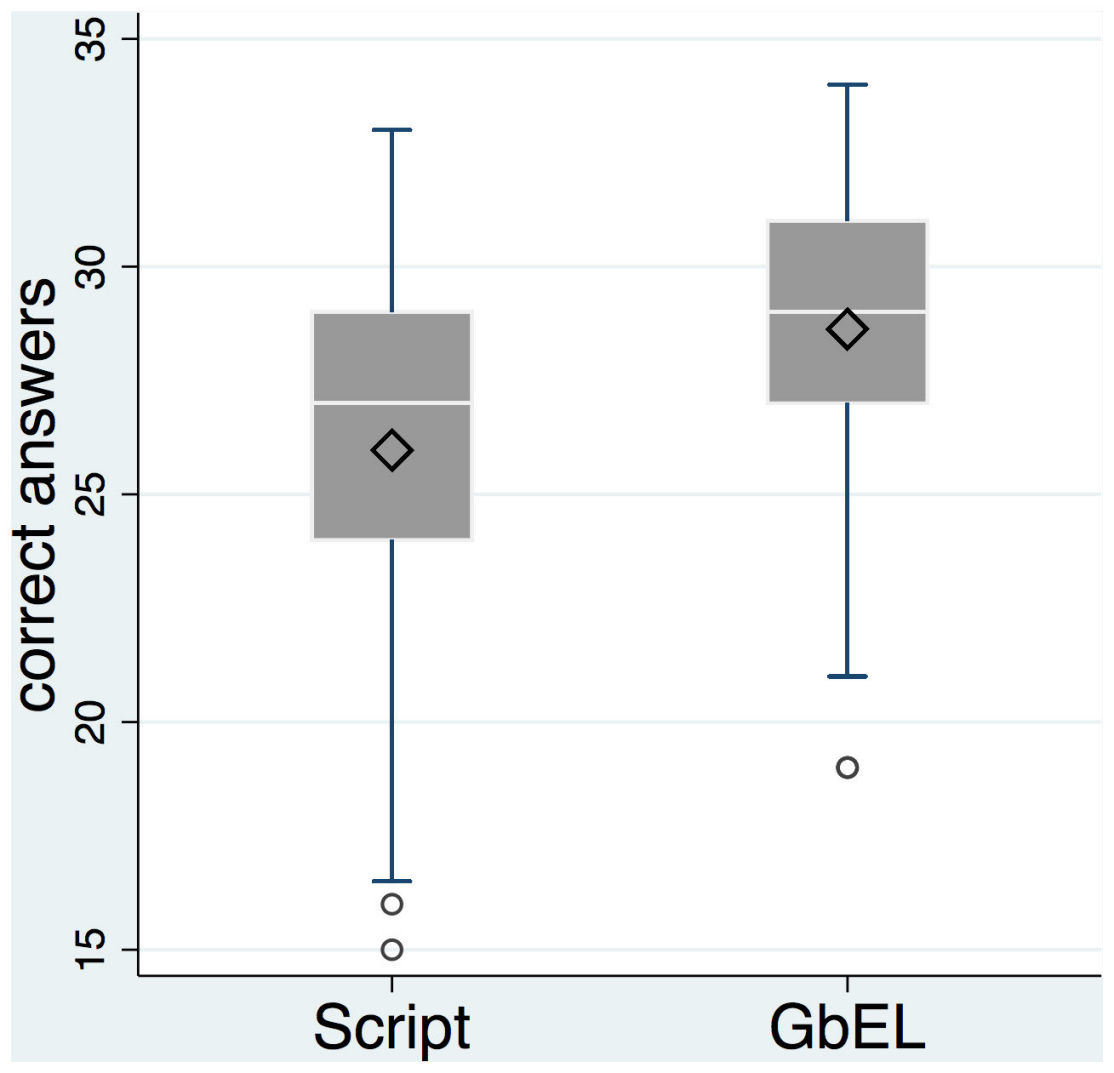
ITT analysis: distribution of the data in the script group and the GbEL group.

The cognitive learning outcome measured with a single-choice test with 34 questions was significantly and effectively higher in the GbEl group when compared with the script group. The means and standard deviations in the ITT analysis were 26.0 (3.99) of a total of 34 points for the script group vs. 28.6 (3.53) in the GbEl group (t-test: p < 0.001) with a Cohen's d effect size of 0.71 (see Figure 4). The difference of 2.66 between the averages of the GbEL group and the script group corresponds to a 7.8 % change on the 34-point scale which is a relevant improvement.

In the ITT-AT analysis, the means were 26.0 for the script group and 29.1 for the GbEl group with a Cohen's d effect size of 0.89. In the ITT-AT analysis the differences between averages of the GbEL group and the script group are even higher: 3.12 points corresponding to 9.2 % change on the 34-point scale. Detailed data is presented in Table 2.

**Table 2:** ITT-analysis and ITT-adjusted-for-treatment (AT) analysis of the cognitive learning performance.

Parameter	Reference	Script	GbEl	difference
N	117	63	81	
gender ratio (M : F)	ND	24 : 39	36 : 45	
Median	17	27	29	2
mean (SD) [SE]	18.3 (4.13)	26.0 (3.99)	28.6 (3.53)	2.66^*^ [0.63]
CI mean	17.5 - 19.0	25.0 - 27.0	27.9 - 29.4	1.42 - 3.909
mean AT [SE]		26.0	29.1	3.12^**^ [0.7]
CI mean AT		25.1 - 26.8	28.2 - 30.0	1.8 - 4.5

Data are shown for the reference group (without specific training) as a baseline measurement and the script group (control) vs. the GbEl group (intervention). Performance was measured with a 34-point single choice test.

The last column shows the performance differences between the GbEl group and the script group. The relevance of the differences should be interpreted considering the baseline value of untrained students from the first column. An increase of 2.66 points from script-based instruction to GbEl is about 35 % of the increase of 7.7 points from baseline to script.

The ITT-AT analysis pronounces the differences between the groups taking into account which students actually received the training. The differences between the groups are highly significant and relevant (7.8 % resp. 9.2 % increase). The Cohen's d effect size is 0.71 for the ITT analysis and 0.89 for the ITT-AT analysis.

^*^: t-test p < 0.001

^**^: Wald test p < 0.001

**Figure 5:**
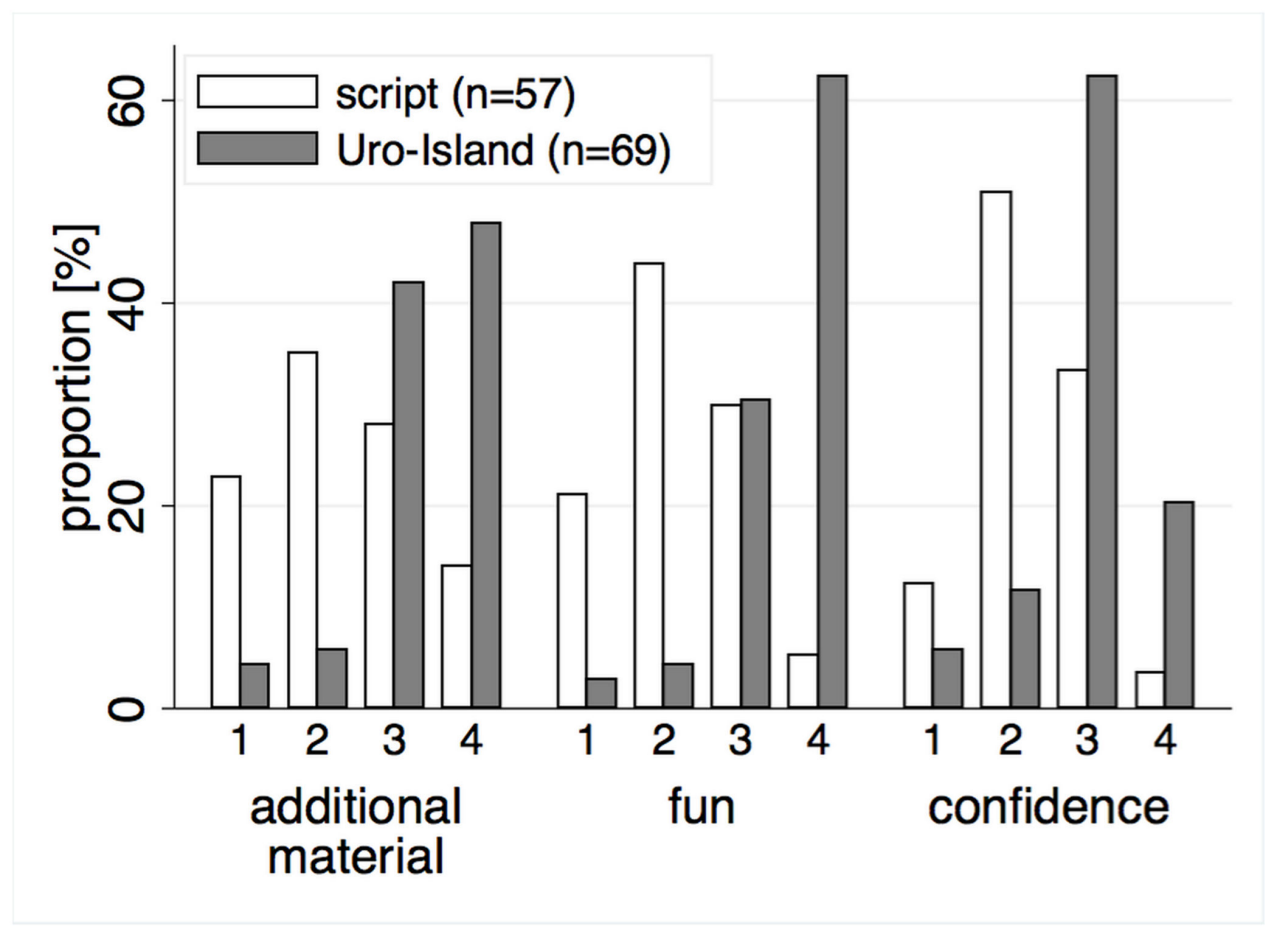
Students' attitudes towards the learning experience, answers on the questionnaire on a 4-point Likert scale (1: no agreement to 4: full agreement). Students in the GbEl group had significantly more fun, would like to learn more with GbEl and felt more confident in the content domain. The following questions were asked: **additional material**: Would you like to have more learning material like this in your training? **fun**: Did you enjoy working with the learning material? **confidence**: Do you feel confident in the domain of laboratory urinalysis now?

Attitudes towards the learning experience measured on a 4-point Likert scale were more positive in the GbEl group than in the script group (see Figure 5). For all three questions, the mean score of the GbEl group was significantly higher. The differences between group means were 1.33 for the question if the students had fun while learning with the respective material, 1.0 for the question if they would like to learn from more of such material, and 0.69 if they felt secure in the topic (Wilcoxon-test: p < 0.001 for all three differences).

## Discussion

In this randomized controlled trial, we have shown that students performed better after GbEl than after a traditional script-based instructional approach. Our study not only shows that students who received the GbEl training had a significantly higher cognitive learning outcome when compared with the students who learned the same material with a script, but also that the former group had more fun, would like to learn more in this style, and are more secure in regard of their knowledge of the topic. With our RCT, we provide clear evidence for a beneficial effect of GbEl on the students' knowledge and their attitudes towards their learning experience.

However, most recent systematic reviews and meta-analysis of studies on Game-based learning and Game-based E-Learning approaches in the medical professions showed limited effects of these instructional methods [3–6]. A 2010 systematic review of Akl et. al. on educational games on medical students' learning outcomes [6] found only five studies with low-to-moderate methodological quality eligible for the analysis. Of these RCTs, three suggested a positive effect of the games on medical students' knowledge. However, the authors concluded, "Due to the limited number of studies, their low-to-moderate methodological quality, and the inconsistent results, the evidence is unlikely to support a general recommendation for the use of educational games in medical schools." With our results presented here, we can positively contribute to the very limited evidence on the effectivity of game-based learning and GbEl.

A major reason for educators to combine gaming with education is the high motivational capacity of games. Activating this potential of enjoyment for education promises effortless learning, even of contents most students do not like to learn. The problem of many educational games, however, is that they easily loose 'gaming character', due to the integration of educational contents and, therefore, their enjoyment and motivational capacity. In this study, we asked students how much fun they had in their learning experience. The gaming group clearly and significantly expressed more fun than the script group. In addition, the GbEl group would have liked to learn more with games than the script group with the scripts. Although we did not *directly* measure motivation and enjoyment with our study, for us, this is one possible explanation for the better cognitive learning results of the GbEl group compared to the script group. We assume that with a higher motivation and more enjoyment, the students in the GbEl group might have spent more time in learning for the specific topics and consequently achieved better cognitive results. Another explanation for better performance is that retention is better in the GbEl group, due to a more active and interactive learning experience.

Although high expectations on GbEl circulate in the educational domain, a number of economical and pragmatic issues of electronic games' production hinder the widespread use of GbEl. On the one hand, it is difficult to represent learning contents and objectives as parts of games without the loss of gaming characteristics and thus the main motivational aspect of gaming. In addition, no widely accepted guidelines exist on how to teach effectively with games and how to implement large quantities of educational material as gaming content. On the other hand, the learning material can only be developed in interdisciplinary teams of domain experts and game developers. Consequently, the development process depending largely on the creativity of the participants can become tedious and expensive.

In this small project without dedicated funding, we have shown that it is possible to create an educational game in a genre which requires some development skills. Inexpensive development frameworks are available for many game genres which abstract the technical implementation from the conceptual development (e.g. [32]). In addition, for most educational games it is not the objective to produce an industrial strength high-fidelity game comparable to major movie productions, from the economical point of view. Therefore, attractive educational games can be produced with small resources. Although generally feasible in smaller educational environments, game development remains work-intensive. Teachers who consider educational games as an interesting instructional alternative should carefully balance benefits and costs prior to project start.

### Limitations

As for limitation of our study, we did not control nor document the time participants were engaged with the learning experience. One could suppose that students in the GbEl group worked significantly longer on the topic than the students in the script group, and so the effect could be due to longer learning activity. Objective time protocoling in the specific application scenario could not be implemented for technical reasons, especially in the script group. Thus, our study is limited in so far that we cannot clearly attribute the positive effects on learning outcome to motivational factors of the instructional method mediated by longer training times or the instructional method itself. However, future studies should differentiate between the effects of motivational factors of the game and its instructional efficacy [43].

In this study, we did not control for certain factors which might have additionally influenced the learning outcome. Specifically, this could have been students practicing urinalysis microscopy outside of the practical-training time on their own. However, on the one hand, this factor is randomly distributed over both groups and, on the other hand, it might be understood as a motivational effect of the specific training, which could not be further analyzed by our study.

Beyond the scope of our study were also the long-term effects of GbEl. As reported, there might be differences between traditional and game-based instruction in long term retention [7]. Due to the anonymized data collection in our study, we were not able to follow up on the students and gather further data on this interesting topic.

Voluntariness of participation can lead to selection bias, if only the active participants are analyzed and if these represent students intrinsically more interested in the topic. We did not question the non-participating students as to why they dropped out and could not follow up on them, due to the same reasons as above. However, we analyzed all students as randomized (ITT). So, we made a fair comparison between offering all students the use of a game or the use of a script. Not unexpectedly, the learning outcome of non-participants was significantly lower than that of students who performed per protocol.

Another limitation of our study is the presentation of the gaming module as an "either/ or" choice, which does not reflect a realistic teaching environment in which the gaming module would be used together with other instructional formats. However, this restriction of our study is a necessary limitation due to the design as a randomized controlled trial in order to attribute a possible effect solely to the interventional method. As evidence for the efficacy of electronic game based learning is rare, this objective had to be our primary objective in this study prior to more generalizable approaches.

### Future Research

Future research questions can be directly taken from the limitations of our study. On the one hand, it would be very important to investigate the direct motivational effects of game-based learning on students in medical training. Another important question is how the long term retention of game-based learning compares with that after training with conventional instructional methods.

To provide evidence on the everyday usability of GbEl, it is not enough to investigate the efficacy of GbEl vs. other instructional methods in a competitive design. Moreover, studies have to be performed which answer the questions regarding what the technology adds to the traditional methods and how they are best integrated into existing curricula. How can GbEl be used in combination with other formats and what will be the costs?

## Conclusions

In a randomized controlled trial, we provided evidence that game-based E-learning results in higher outcome performance of students compared to a traditional script-based instructional approach. Our study not only shows that students who received the GbEl training had a significantly higher cognitive learning outcome when compared with the students who learned the same material with a script but had more fun, would like to learn more in this style and are more secure in regard of their knowledge of the topic.

Game-based E-learning should be taken seriously into account as an alternative instructional method on topics where student motivation might be a problem.
